# Supplementary material for: Modeling glioblastoma heterogeneity as a dynamic network of cell states
Source: Mol Syst Biol. 2021 Sep 16;17(9):e10105. doi: 10.15252/msb.202010105 (PMC8444284; doi:10.15252/msb.202010105)
Supplement: Supplementary file 6 — Source Data for Figure 5 [file MSB-17-e10105-s004.zip › Figure5A_sourcedata/GSEA_3071/nosigdb_stateIII.GseaPreranked.1622795155562/RICHARDS_NOWAKOWSKI_SCIENCE_2017_RG_EARLY_UPREG.html]

Details for gene set RICHARDS\_NOWAKOWSKI\_SCIENCE\_2017\_RG\_EARLY\_UPREG[GSEA]

|  || Dataset | state33071 |
| Phenotype | NoPhenotypeAvailable |
| Upregulated in class | na\_pos |
| GeneSet | RICHARDS\_NOWAKOWSKI\_SCIENCE\_2017\_RG\_EARLY\_UPREG |
| Enrichment Score (ES) | 0.36824524 |
| Normalized Enrichment Score (NES) | NaN |
| Nominal p-value | NaN |
| FDR q-value | 1.0 |
| FWER p-Value | 0.0 |
Table: GSEA Results Summary

  

Fig 1: Enrichment plot: RICHARDS\_NOWAKOWSKI\_SCIENCE\_2017\_RG\_EARLY\_UPREG      
 Profile of the Running ES Score & Positions of GeneSet Members on the Rank Ordered List

  

| PROBE | GENE SYMBOL | GENE\_TITLE | RANK IN GENE LIST | RANK METRIC SCORE | RUNNING ES | CORE ENRICHMENT || 1 | HSPB1 |  |  | 4 | 0.650 | 0.0069 | Yes |
| 2 | RPLP0 |  |  | 6 | 0.633 | 0.0145 | Yes |
| 3 | RPS12 |  |  | 8 | 0.615 | 0.0218 | Yes |
| 4 | RPS26 |  |  | 9 | 0.595 | 0.0291 | Yes |
| 5 | RPL12 |  |  | 12 | 0.579 | 0.0357 | Yes |
| 6 | RPL6 |  |  | 16 | 0.555 | 0.0417 | Yes |
| 7 | RPL35 |  |  | 17 | 0.549 | 0.0485 | Yes |
| 8 | RPS25 |  |  | 19 | 0.527 | 0.0547 | Yes |
| 9 | RPL37 |  |  | 20 | 0.526 | 0.0612 | Yes |
| 10 | RPL24 |  |  | 21 | 0.524 | 0.0677 | Yes |
| 11 | RPL19 |  |  | 24 | 0.517 | 0.0735 | Yes |
| 12 | RPLP1 |  |  | 25 | 0.514 | 0.0798 | Yes |
| 13 | RPL23A |  |  | 27 | 0.502 | 0.0858 | Yes |
| 14 | RPLP2 |  |  | 36 | 0.489 | 0.0896 | Yes |
| 15 | POLR2L |  |  | 38 | 0.482 | 0.0953 | Yes |
| 16 | RPS21 |  |  | 39 | 0.482 | 0.1012 | Yes |
| 17 | RPS6 |  |  | 41 | 0.480 | 0.1069 | Yes |
| 18 | RPL14 |  |  | 42 | 0.479 | 0.1128 | Yes |
| 19 | RPL8 |  |  | 44 | 0.469 | 0.1183 | Yes |
| 20 | RPL13A |  |  | 45 | 0.467 | 0.1241 | Yes |
| 21 | RPL5 |  |  | 51 | 0.461 | 0.1284 | Yes |
| 22 | NACA |  |  | 53 | 0.459 | 0.1338 | Yes |
| 23 | NDUFA6 |  |  | 54 | 0.458 | 0.1394 | Yes |
| 24 | RPL32 |  |  | 55 | 0.457 | 0.1450 | Yes |
| 25 | RPL18A |  |  | 58 | 0.453 | 0.1501 | Yes |
| 26 | CYCS |  |  | 59 | 0.453 | 0.1557 | Yes |
| 27 | RPS18 |  |  | 61 | 0.451 | 0.1610 | Yes |
| 28 | RPS24 |  |  | 69 | 0.444 | 0.1646 | Yes |
| 29 | RPL30 |  |  | 71 | 0.442 | 0.1697 | Yes |
| 30 | RPL39 |  |  | 72 | 0.442 | 0.1752 | Yes |
| 31 | RPS28 |  |  | 73 | 0.440 | 0.1806 | Yes |
| 32 | RPS7 |  |  | 75 | 0.436 | 0.1857 | Yes |
| 33 | SLIRP |  |  | 80 | 0.430 | 0.1899 | Yes |
| 34 | RPS3A |  |  | 82 | 0.429 | 0.1950 | Yes |
| 35 | RPS19 |  |  | 84 | 0.427 | 0.1999 | Yes |
| 36 | YWHAE |  |  | 85 | 0.427 | 0.2052 | Yes |
| 37 | NPM1 |  |  | 88 | 0.421 | 0.2099 | Yes |
| 38 | RPS3 |  |  | 90 | 0.420 | 0.2148 | Yes |
| 39 | RPL10A |  |  | 94 | 0.414 | 0.2191 | Yes |
| 40 | RPS27A |  |  | 95 | 0.413 | 0.2242 | Yes |
| 41 | RPF2 |  |  | 101 | 0.410 | 0.2279 | Yes |
| 42 | TOMM5 |  |  | 103 | 0.409 | 0.2326 | Yes |
| 43 | EIF3J |  |  | 110 | 0.403 | 0.2360 | Yes |
| 44 | SERF2 |  |  | 111 | 0.403 | 0.2409 | Yes |
| 45 | PPIA |  |  | 121 | 0.395 | 0.2434 | Yes |
| 46 | SNRPB |  |  | 122 | 0.395 | 0.2483 | Yes |
| 47 | NOL7 |  |  | 126 | 0.392 | 0.2523 | Yes |
| 48 | RPS23 |  |  | 129 | 0.391 | 0.2566 | Yes |
| 49 | GNG5 |  |  | 139 | 0.382 | 0.2589 | Yes |
| 50 | RPL21 |  |  | 141 | 0.382 | 0.2633 | Yes |
| 51 | RPL13 |  |  | 152 | 0.372 | 0.2652 | Yes |
| 52 | GLO1 |  |  | 161 | 0.367 | 0.2676 | Yes |
| 53 | CACYBP |  |  | 165 | 0.364 | 0.2713 | Yes |
| 54 | PSMB3 |  |  | 171 | 0.361 | 0.2744 | Yes |
| 55 | RPL18 |  |  | 178 | 0.355 | 0.2772 | Yes |
| 56 | RPS15 |  |  | 180 | 0.353 | 0.2812 | Yes |
| 57 | FTH1 |  |  | 181 | 0.352 | 0.2856 | Yes |
| 58 | SNRPG |  |  | 186 | 0.349 | 0.2888 | Yes |
| 59 | RPS27L |  |  | 188 | 0.348 | 0.2928 | Yes |
| 60 | RPS14 |  |  | 196 | 0.340 | 0.2951 | Yes |
| 61 | RPL29 |  |  | 201 | 0.338 | 0.2982 | Yes |
| 62 | HINT1 |  |  | 205 | 0.335 | 0.3016 | Yes |
| 63 | GSTP1 |  |  | 211 | 0.333 | 0.3043 | Yes |
| 64 | POLR2J |  |  | 215 | 0.331 | 0.3076 | Yes |
| 65 | NDUFB1 |  |  | 224 | 0.326 | 0.3095 | Yes |
| 66 | RPS2 |  |  | 228 | 0.325 | 0.3127 | Yes |
| 67 | ENO1 |  |  | 241 | 0.314 | 0.3133 | Yes |
| 68 | SRP14 |  |  | 247 | 0.311 | 0.3158 | Yes |
| 69 | RPS8 |  |  | 248 | 0.311 | 0.3196 | Yes |
| 70 | RPL23 |  |  | 250 | 0.310 | 0.3232 | Yes |
| 71 | PTGES3 |  |  | 252 | 0.310 | 0.3267 | Yes |
| 72 | RPL7A |  |  | 255 | 0.308 | 0.3300 | Yes |
| 73 | RPL41 |  |  | 265 | 0.303 | 0.3313 | Yes |
| 74 | SNHG6 |  |  | 266 | 0.303 | 0.3351 | Yes |
| 75 | LSM4 |  |  | 268 | 0.303 | 0.3385 | Yes |
| 76 | RPL9 |  |  | 269 | 0.302 | 0.3423 | Yes |
| 77 | RBM8A |  |  | 276 | 0.298 | 0.3443 | Yes |
| 78 | HSPE1 |  |  | 279 | 0.297 | 0.3475 | Yes |
| 79 | UBB |  |  | 294 | 0.293 | 0.3473 | Yes |
| 80 | TMEM258 |  |  | 295 | 0.292 | 0.3509 | Yes |
| 81 | HMGB1 |  |  | 318 | 0.286 | 0.3486 | Yes |
| 82 | GAPDH |  |  | 320 | 0.286 | 0.3518 | Yes |
| 83 | TPT1 |  |  | 324 | 0.285 | 0.3545 | Yes |
| 84 | SNRPE |  |  | 332 | 0.283 | 0.3561 | Yes |
| 85 | CSNK2B |  |  | 333 | 0.283 | 0.3596 | Yes |
| 86 | MYL6 |  |  | 360 | 0.272 | 0.3560 | Yes |
| 87 | ZFAND2A |  |  | 361 | 0.272 | 0.3594 | Yes |
| 88 | RPL15 |  |  | 363 | 0.271 | 0.3624 | Yes |
| 89 | SNRPD1 |  |  | 376 | 0.268 | 0.3625 | Yes |
| 90 | H2AFZ |  |  | 386 | 0.265 | 0.3634 | Yes |
| 91 | RAN |  |  | 388 | 0.264 | 0.3664 | Yes |
| 92 | RPL7 |  |  | 394 | 0.262 | 0.3682 | Yes |
| 93 | PSMB7 |  |  | 412 | 0.256 | 0.3669 | No |
| 94 | HK2 |  |  | 432 | -0.250 | 0.3648 | No |
| 95 | ATF3 |  |  | 515 | -0.255 | 0.3460 | No |
| 96 | PHLDA1 |  |  | 589 | -0.259 | 0.3297 | No |
| 97 | GPC4 |  |  | 593 | -0.259 | 0.3321 | No |
| 98 | PLAG1 |  |  | 623 | -0.261 | 0.3275 | No |
| 99 | NDC80 |  |  | 636 | -0.261 | 0.3275 | No |
| 100 | MCM4 |  |  | 638 | -0.261 | 0.3305 | No |
| 101 | ZFP36L2 |  |  | 671 | -0.263 | 0.3251 | No |
| 102 | CBWD2 |  |  | 762 | -0.267 | 0.3043 | No |
| 103 | CCDC181 |  |  | 797 | -0.269 | 0.2985 | No |
| 104 | SERTAD4 |  |  | 849 | -0.272 | 0.2882 | No |
| 105 | HELLS |  |  | 970 | -0.278 | 0.2595 | No |
| 106 | MCM3 |  |  | 1021 | -0.280 | 0.2496 | No |
| 107 | CDCA3 |  |  | 1029 | -0.281 | 0.2512 | No |
| 108 | BOC |  |  | 1034 | -0.281 | 0.2536 | No |
| 109 | TRIM59 |  |  | 1110 | -0.285 | 0.2370 | No |
| 110 | PCNA |  |  | 1116 | -0.286 | 0.2392 | No |
| 111 | EFNB1 |  |  | 1198 | -0.290 | 0.2210 | No |
| 112 | NUSAP1 |  |  | 1262 | -0.293 | 0.2078 | No |
| 113 | AGO3 |  |  | 1268 | -0.293 | 0.2101 | No |
| 114 | LINC00339 |  |  | 1294 | -0.295 | 0.2070 | No |
| 115 | PHLPP1 |  |  | 1317 | -0.296 | 0.2047 | No |
| 116 | ZNF516 |  |  | 1360 | -0.298 | 0.1972 | No |
| 117 | KCTD6 |  |  | 1392 | -0.299 | 0.1926 | No |
| 118 | CCNL1 |  |  | 1411 | -0.301 | 0.1914 | No |
| 119 | CDON |  |  | 1423 | -0.301 | 0.1922 | No |
| 120 | CREB5 |  |  | 1470 | -0.304 | 0.1836 | No |
| 121 | KIF4A |  |  | 1504 | -0.306 | 0.1786 | No |
| 122 | TGIF1 |  |  | 1506 | -0.306 | 0.1821 | No |
| 123 | ZEB1 |  |  | 1569 | -0.310 | 0.1693 | No |
| 124 | PPM1D |  |  | 1579 | -0.311 | 0.1707 | No |
| 125 | ALDH1B1 |  |  | 1630 | -0.314 | 0.1612 | No |
| 126 | PDK1 |  |  | 1723 | -0.320 | 0.1405 | No |
| 127 | ZNF844 |  |  | 1730 | -0.321 | 0.1429 | No |
| 128 | HAUS8 |  |  | 1741 | -0.321 | 0.1441 | No |
| 129 | EAF1 |  |  | 1746 | -0.321 | 0.1470 | No |
| 130 | PEG3 |  |  | 1754 | -0.322 | 0.1491 | No |
| 131 | GLI2 |  |  | 1843 | -0.327 | 0.1296 | No |
| 132 | BRCA1 |  |  | 1844 | -0.327 | 0.1336 | No |
| 133 | TAF8 |  |  | 1892 | -0.329 | 0.1251 | No |
| 134 | NEK4 |  |  | 1929 | -0.331 | 0.1195 | No |
| 135 | UBE2C |  |  | 1942 | -0.332 | 0.1204 | No |
| 136 | SALL1 |  |  | 1962 | -0.333 | 0.1194 | No |
| 137 | SMC2 |  |  | 1963 | -0.333 | 0.1235 | No |
| 138 | RDH10 |  |  | 2051 | -0.338 | 0.1044 | No |
| 139 | MXD1 |  |  | 2053 | -0.338 | 0.1083 | No |
| 140 | RRM2 |  |  | 2147 | -0.345 | 0.0876 | No |
| 141 | TNFRSF19 |  |  | 2165 | -0.346 | 0.0873 | No |
| 142 | MFAP2 |  |  | 2169 | -0.347 | 0.0908 | No |
| 143 | H1F0 |  |  | 2207 | -0.349 | 0.0852 | No |
| 144 | TPX2 |  |  | 2289 | -0.356 | 0.0679 | No |
| 145 | HMGA2 |  |  | 2330 | -0.360 | 0.0616 | No |
| 146 | TTYH1 |  |  | 2397 | -0.365 | 0.0484 | No |
| 147 | DLGAP5 |  |  | 2421 | -0.367 | 0.0468 | No |
| 148 | IQGAP3 |  |  | 2592 | -0.381 | 0.0060 | No |
| 149 | HEY1 |  |  | 2593 | -0.381 | 0.0107 | No |
| 150 | PAX6 |  |  | 2619 | -0.384 | 0.0087 | No |
| 151 | CCNA2 |  |  | 2635 | -0.385 | 0.0094 | No |
| 152 | MDM1 |  |  | 2643 | -0.385 | 0.0123 | No |
| 153 | KIF20B |  |  | 2689 | -0.390 | 0.0050 | No |
| 154 | NOTCH3 |  |  | 2759 | -0.395 | -0.0086 | No |
| 155 | HSPA1A |  |  | 2774 | -0.396 | -0.0074 | No |
| 156 | ABHD3 |  |  | 2805 | -0.400 | -0.0105 | No |
| 157 | SFRP1 |  |  | 2839 | -0.402 | -0.0144 | No |
| 158 | PER2 |  |  | 2864 | -0.404 | -0.0159 | No |
| 159 | ATP1B1 |  |  | 2865 | -0.405 | -0.0109 | No |
| 160 | SMC4 |  |  | 2872 | -0.405 | -0.0075 | No |
| 161 | ITGB8 |  |  | 3026 | -0.424 | -0.0433 | No |
| 162 | SLC3A2 |  |  | 3063 | -0.427 | -0.0476 | No |
| 163 | FANCD2 |  |  | 3065 | -0.427 | -0.0426 | No |
| 164 | TIPARP |  |  | 3072 | -0.428 | -0.0390 | No |
| 165 | JAM3 |  |  | 3096 | -0.430 | -0.0398 | No |
| 166 | ATAD2 |  |  | 3115 | -0.433 | -0.0393 | No |
| 167 | SEMA5A |  |  | 3229 | -0.447 | -0.0641 | No |
| 168 | MCAM |  |  | 3353 | -0.472 | -0.0912 | No |
| 169 | KIF14 |  |  | 3384 | -0.476 | -0.0934 | No |
| 170 | ZFP36L1 |  |  | 3487 | -0.503 | -0.1145 | No |
| 171 | NCAPG |  |  | 3513 | -0.512 | -0.1149 | No |
| 172 | SYNE2 |  |  | 3523 | -0.518 | -0.1109 | No |
| 173 | ZFHX4 |  |  | 3540 | -0.524 | -0.1087 | No |
| 174 | CDC25C |  |  | 3551 | -0.526 | -0.1049 | No |
| 175 | SOX9 |  |  | 3560 | -0.531 | -0.1005 | No |
| 176 | CKB |  |  | 3572 | -0.536 | -0.0968 | No |
| 177 | DACH1 |  |  | 3607 | -0.553 | -0.0991 | No |
| 178 | SOX2 |  |  | 3613 | -0.555 | -0.0936 | No |
| 179 | ANLN |  |  | 3625 | -0.562 | -0.0897 | No |
| 180 | HIST1H1D |  |  | 3637 | -0.569 | -0.0856 | No |
| 181 | SLC5A3 |  |  | 3647 | -0.575 | -0.0809 | No |
| 182 | TOP2A |  |  | 3671 | -0.594 | -0.0798 | No |
| 183 | FAT1 |  |  | 3685 | -0.604 | -0.0758 | No |
| 184 | CENPE |  |  | 3711 | -0.625 | -0.0748 | No |
| 185 | STON2 |  |  | 3739 | -0.658 | -0.0739 | No |
| 186 | HIST1H1B |  |  | 3749 | -0.669 | -0.0681 | No |
| 187 | HSP90B1 |  |  | 3757 | -0.681 | -0.0616 | No |
| 188 | ADAMTS9 |  |  | 3761 | -0.687 | -0.0539 | No |
| 189 | MKI67 |  |  | 3788 | -0.721 | -0.0520 | No |
| 190 | CENPF |  |  | 3842 | -0.820 | -0.0561 | No |
| 191 | PROM1 |  |  | 3854 | -0.846 | -0.0486 | No |
| 192 | SDK2 |  |  | 3868 | -0.898 | -0.0410 | No |
| 193 | C1orf61 |  |  | 3879 | -0.986 | -0.0315 | No |
| 194 | ID4 |  |  | 3893 | -1.061 | -0.0219 | No |
| 195 | ASPM |  |  | 3899 | -1.166 | -0.0089 | No |
| 196 | HIST1H4C |  |  | 3914 | -1.352 | 0.0040 | No |
Table: GSEA details [plain text format]

  

Fig 2: RICHARDS\_NOWAKOWSKI\_SCIENCE\_2017\_RG\_EARLY\_UPREG: Random ES distribution      
 Gene set null distribution of ES for **RICHARDS\_NOWAKOWSKI\_SCIENCE\_2017\_RG\_EARLY\_UPREG**
